# Biocompatibility of Zinc Matrix Biodegradable Composites Reinforced by Graphene Nanosheets

**DOI:** 10.3390/ma15186481

**Published:** 2022-09-19

**Authors:** Mei Fan, Fei Zhao, Shanshan Peng, Qianfei Dai, Yuan Liu, Sheng Yin, Zongkui Zhang

**Affiliations:** 1 College of Materials and Metallurgy, Guizhou University, Guiyang 550025, China; 2 Key Laboratory for Materials Structure and Strength of Guizhou Province, Guiyang 550025, China; 3 Hospital of Guizhou University, Guiyang 550025, China

**Keywords:** zinc matrix composites, cell compatibility, blood compatibility, biodegradable material

## Abstract

As a new type of biodegradable implant material, zinc matrix composites have excellent potential in the application of biodegradable implants because of their better corrosion resistance than magnesium matrix materials. Our previous studies have shown that graphene nanosheet reinforced zinc matrix composites (Zn-GNS) prepared by spark plasma sintering (SPS) have good mechanical properties and suitable degradation rate. However, the biocompatibility of zinc matrix composites is still a problem of concern. The cytocompatibility and blood compatibility of pure zinc and Zn-GNS composites in vitro were studied. The results showed that Zn-GNS composites had acceptable toxicity to MG-63 human osteosarcoma cells. In addition, the hemolysis rate of pure zinc and its composites were less than 3%, which has no adverse effect on adhered platelets, and has good antithrombotic and antiadhesion platelets properties. In conclusion, the addition of GNS did not adversely affect the biocompatibility of Zn-GNS composites, which indicated that Zn-GNS composites are a promising candidate for bone implantation.

## 1. Introduction

Zinc matrix materials have been considered as potential biodegradable implant materials due to their good biocompatibility [1,2,3,4,5,6] and suitable corrosion properties [1,5,6,7]. In addition, zinc is one of the essential trace elements for human body. It participates in the synthesis of most enzymes in the body and plays a vital role in important physiological processes such as human growth and development, reproductive genetics, immune endocrine etc., [8,9]. More importantly, zinc plays an important role in stimulating the proliferation of osteoblasts and preserving bone mass [10,11,12]. However, as-cast pure zinc has poor mechanical properties, with tensile strength of about 20 MPa and elongation of about 0.2% [13], which is far from reaching the strength requirements as orthopedic implant material (the tensile strength is greater than 200 MPa, The elongation is greater than 10% [14]). Alloying is one of the main methods to improve the mechanical strength of zinc alloys. For example, Tang et al. [15]. added 1 wt% Mg to Zn-3Cu alloy, and the yield strength of the alloy increased from 213.7 MPa to 426.7 MPa, but the fracture elongation decreased rapidly from 47.1% to 0.9%. Li et al. [16]. reported that the addition of alloying elements Mg, Ca, and Sr can significantly improve the tensile strength of zinc alloys, but the elongation is poor (less than 10%). The above studies show that although alloying improves the strength of zinc alloys, the plasticity will be greatly reduced, which limits the application of the material. In view of this, nano reinforced materials have received increasing attention in improving the mechanical properties of Zn matrix composites.

Graphene nanosheets (GNS) are composed of a small number of graphene sheets bonded together by van der Waals force and covalent bonds of adjacent carbon atoms which have excellent mechanical strength (130 GPa) and shows good biocompatibility in contact with blood [17,18]. The reduction of GO into RGO reduces the water dispersibility due to fewer oxygen-containing functional groups, thereby stabilizing GO in the body and minimizing its possible cytotoxicity [19,20]. Generally, the biosafety and stability of RGO is superior to that of GO in vivo [20]. However, RGO lacks functional or biochemical groups that might stimulate osteogenesis [21]. GNPs are mostly biodegraded in the physiological environments may occur via enzymatic oxidation by peroxidase [22]. Furthermore, its large surface areas can facilitate the binding of biomolecules on the surface, enhancing its ability to interact with the surrounding environment [23,24]. Several studies have shown that GNPs is biocompatible. For example, Munir et al. [25] reported magnesium-graphene nanosheets (Mg-GNPs) composites and found that the addition of GNPs could significantly improve the viability of SaOS2 cells. Similarly, Saberi et al. [26] reported that lower concentrations (0.5–1 wt%) of GNPs did not produce any toxic response to the Mg-GNPs composite and instead led to the adhesion and proliferation of MG-63 cells.

Our group has studied the mechanical properties and in vitro biodegradability of rolled Zn-GNS composites [27]. When the GNS content was 0.7 wt%, its tensile strength, elongation, and degradation rates were 254 MPa, 16.7%, and 301 μm/a, respectively, which fully meets the standards of biodegradable metal implants. In addition, Zn-GNS composites as a biodegradable implant material must be tested for its possible adverse effects to cell growth. However, the biocompatibility of Zn-GNS composites was not studied in previous papers. Therefore, in this study, we studied the in vitro biocompatibility of Zn-GNS composites through cytotoxicity test, hemolysis rate test and activated partial thromboplastin time test, and contact angle test.

## 2. Materials and Methods

### 2.1. Material Preparation 

Zinc powder with an average grain size of 5 µm (99.5% purity) (Beijing Zhongnuo New Material Technology Co., Ltd., Beijing, China) and GNS with a thickness of 1–3 layers (0.686–1.054 nm) (Shenzhen Zhongsen Linghang Technology Co., Ltd., Shenzhen, China) as the raw material. Pure zinc and Zn-xGNS (x = 0.3 and 0.7 wt%) composites were prepared by powder metallurgy. The detailed preparation process has been described in our previous study [26]. In short, GNS was modified by sodium dodecyl sulfate (SDS) surfactant, and then the modified GNS and zinc powder were ball milled at 300 r/min for 6 h in planet ball mill (QM-3SP2), and absolute ethanol was added as a control agent. After ball milling, the uniformly mixed powder was dried in vacuum for 8 h, and then sintered by spark plasma sintering (SPS-625HF) under the process of sintering temperature of 340 °C, sintering pressure of 45 MPa and holding time of 10 min. The size of the sample was Φ 60 mm × 7 mm cylindrical material. Then, the mechanical properties of the material are improved by multi-pass hot deformation rolling process. Pure Zn prepared under the same process was used as a control.

### 2.2. Extraction Preparation 

A block sample with a size of 0.5 cm × 0.5 cm × 0.1 cm was cut by electrical discharge machining to study the biocompatibility. The sample was sterilized under high temperature and high pressure at 121 °C for 20 min. The extract was prepared according to ISO10993-5:2009 [28]. The sample was immersed in MEM medium (10% fetal bovine serum) with a ratio of 1.25 cm^2^/mL for 24 h at 37 °C, and filtered by a 0.22 um microporous membrane. The extract was then diluted with concentrations of 5%, 10%, and 15% for the following tests.

### 2.3. Cell Viability Test

The cytocompatibility of biomaterials can be better distinguished by selecting appropriate cell types for cytotoxicity evaluation according to the application sites of medical devices. In this paper, the Zn-GNS composites is a medical material used in orthopedic implant, which must be in contact with bone cells when implanted into the bone environment, and MG-63 cells are human osteosarcoma cells. Therefore, MG-63 cells were selected for cytotoxicity test in this study. Previous studies reported that most zinc matrix biodegradable materials also selected MG-63 cells for cell viability testing [29,30,31]. First, MG-63 cells were seeded in 96-well plates at a density of 4 × 10^4^ cells/well and cultured until the cells adhered, then the diluted extract was used to replace the culture medium, and the culture plate was cultured at 37 °C in a humidified atmosphere of 5% CO_2_. After 24 h, 48 h, and 72 h, 100 μL of CCK-8 solution was added to each well and cultured for 2 h. Normally cultured cells were used as the control group. The absorbance (OD value) at 450 nm was detected by microplate reader (TECAN, SPARK 10 M). The calculation of relative growth rate is as follows:

(1) $$\mathrm{RGR}\left(\%\right)=\frac{OD_{sample}-OD_{blank\;}}{OD_{Control}-OD_{blank}}\times 100\%$$

Live/dead staining experiments were performed on MG-63 cells co-cultured with 5% extract at different times. Calcein-AM and propidium iodide staining solution were added to the well plate and incubated for 15 min in the dark. The cells were washed in the well plate with PBS to remove excess serum. The cell morphology was observed with a confocal microscope (Olympus, Tokyo, Japan, FV1200).

### 2.4. Hemolysis Test

The blood of healthy volunteers was added with 3.2% sodium citrate anticoagulant and the blood was diluted to obtain red blood cell suspension. The prepared extract (5% and 10%) was added to a centrifuge tube containing 0.2 mL red blood cell suspension, incubated at 37 °C for 60 min and centrifuged for 5 min. Deionized water and normal saline were used for the positive and negative control groups, respectively. About 0.2 mL of the supernatant was transferred to a 96-well plate, then the absorbance (OD value) was measured by a microplate reader (TECAN, SPARK 10 M) at a wavelength of 545 nm. Three parallel experiments were performed on each sample. The hemolysis rate of the sample is calculated according to the following formula:

(2) $$\mathrm{Hemolysis}\;\left(\%\right)=\frac{D_{t}-D_{nc}}{D_{pc}-D_{nc}}\times 100\%$$

Among them, *D_t_* is the absorbance of the experimental sample; *D_nc_* is the absorbance of the negative control group; *D_pc_* is the absorbance of the positive control group. 

### 2.5. Platelet Adhesion Test

A total of 36 uL of platelet-rich plasma prepared by hemolysis test and 4 uL of extract with a concentration of 5% were taken in a centrifuge tube, and incubated at 37 °C for 30 min. Then 10 uL of platelet-rich plasma drop was placed on the surface of a clean glass slide for 60 min. Then, the poorly adhered platelets were washed with normal saline several times, and the adhered platelets were fixed with 4% paraformaldehyde dropwise at room temperature for 10 min. After this time, rinse three times with normal saline, rinse 1 time with distilled water, and dry them naturally. The morphology of the adhered platelets on the glass surface was observed by laser three-dimensional microscopic imaging system (VK-150K, Japan Keyence, Osaka, Japan). The same volume of platelet rich plasma and normal saline was added as the control.

### 2.6. Coagulation Time

The anticoagulated whole blood was centrifuged at 3000 rpm for 10 min to collect upper platelet poor plasma (PPP). A total of 360 uL of the above prepared plasma and 40 uL of the extract with a concentration of 5% were taken in a 15 mL centrifuge tube, and allowed to sit at 37 °C for 30 min. The APTT value was measured by an automatic coagulation analyzer (CS-5100, USA Sysmex, Baltimore, MD, USA). About 360 uL of platelet poor plasma was added to 40 uL of normal saline as the experimental control.

### 2.7. Contact Angle Measurement

At room temperature, a contact angle goniometer (JC2000D1) was used to measure the contact angle of pure Zn and Zn-GNS composites. A micro syringe was used to evenly drop 2 mL of the simulated body fluid (SBF) onto the surface of the material. After that, an image of the droplet is captured. For the average value, each specimen was measured three times.

## 3. Results and Discussion

### 3.1. Cytotoxicity of Zinc-Based Composites

**Figure 1:**
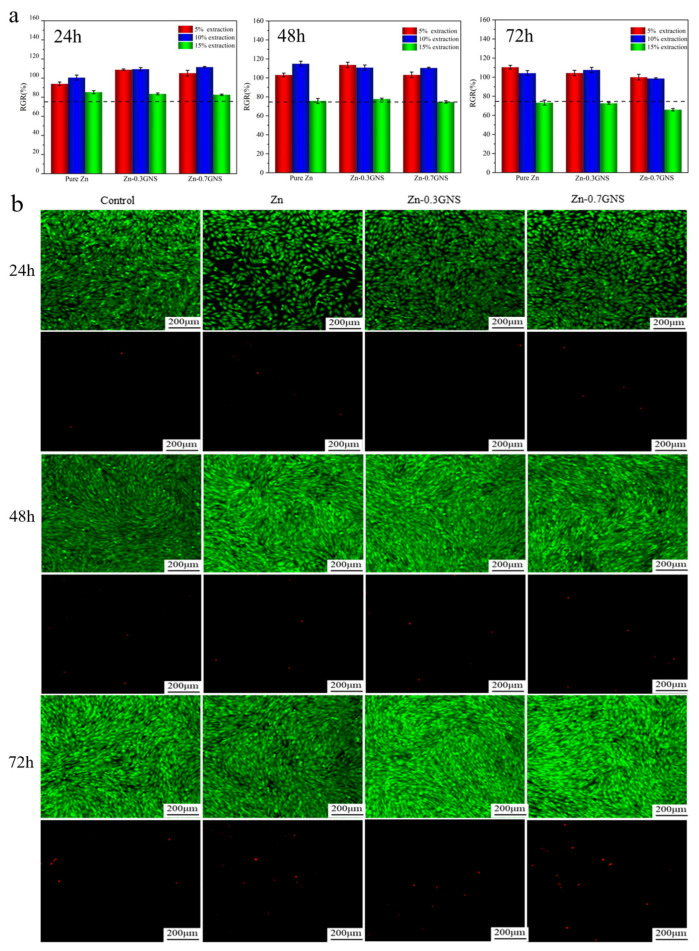
Cytocompatibility of pure Zn and Zn-GNS composites. (**a**) relative activity of cells; (**b**) morphology of MG-63 cells cultured in 5% extract for 24 h, 48 h and 72 h. Green represents living cells and red represents dead cells.

Figure 1a shows the relative activity of human osteosarcoma MG-63 cells cultured in 5%, 10%, and 15% extracts of pure Zn and Zn-GNS composites for 24 h, 48 h, and 72 h, respectively. The results showed that the concentration of the extract affected the relative activity of the cells. It can be observed that the relative activity of cells in 5% and 10% concentration of pure Zn and Zn-GNS composites extract is greater than 90%, and even the proliferation rate is higher than 100% which promotes the growth and division of cells. According to ISO10993-5-2009 cytotoxicity evaluation standard (RGR > 75%), it shows good biocompatibility. Saberi et al. [26] reported the biological properties of graphene nano-platelet reinforced magnesium matrix composites (Mg-xGNP (x = 0.5, 0.1 wt%)). Compared with pure magnesium, the addition of GNP can significantly improve the cell viability and adhesion of the composite to MG-63 cells, and promote the osteogenic differentiation of MG-63 cells. It shows that the addition of GNS does not adversely affect the cytocompatibility. The 15% extract had a certain inhibitory effect on cell survival, which may be attributed to the high concentration of zinc ions in the extract. This has been reported by Murni [32]. In addition, Wang et al. [33] believed that due to the huge difference between in vitro and in vivo conditions, the differences in sensitivities of cells to in vitro and in vivo degradable ions and the capability of in vivo circulation system to dilute local degradation products were fully considered in the study of in vitro cytotoxicity test. It was suggested to use the extract diluted 6 to 10 times to test biodegradable metals. On the other hand, according to our previous research results, the degradation rates of Zn-xGNS composites are 0.201 and 0.301 mm/a, respectively. Taking the boundary dimension of a medical PLLA screw into consideration, its surface area is approximately 2.0 cm^2^ [34]; the zinc ion release rates of Zn-GNS composites screws with the same surface area were 0.772 and 1.152 mg/d, respectively. It is far lower than the daily intake of zinc in healthy adults (15–40 mg/d) [35]. Therefore, the biosafety of Zn-GNS composites will be guaranteed.

Figure 1b shows the fluorescence images of MG-63 cells cultured in 5% extract of Zn-GNS composites for 24 h, 48 h, and 72 h respectively. It can be seen that the morphology of living cells is mostly long spindle, rhombic, and polygonal, and the number of live cells is larger than that of dead. After 72 h of culture, the number of living cells increases significantly, even became crowded in the field of view, and the number of dead cells does not change significantly. Compared with the cell morphology and cell number of the control group, it can be shown that MG-63 cells can grow on the surface of the Zn-GNS composites, and Zn-GNS composites have excellent cytocompatibility. This result further verified the above cytotoxicity experiment.

### 3.2. Blood Compatibility of Zinc Matrix Composites

#### 3.2.1. Hemolysis Rate

**Figure 2:**
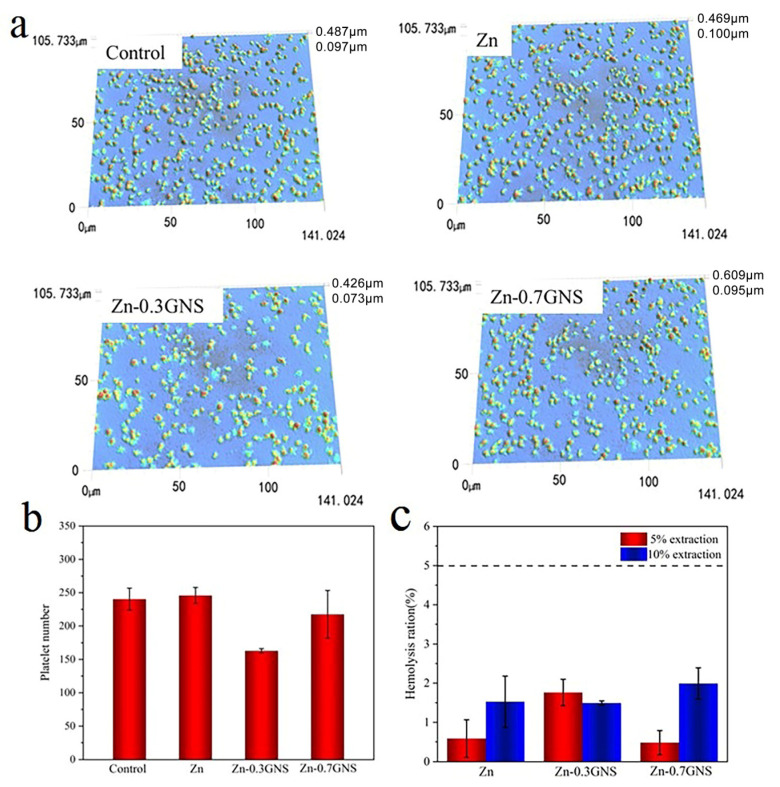
(**a**) 3D platelets adhesion morphology, (**b**) platelets adhesion number, and (**c**) hemolysis percentage of pure Zn and Zn-GNS composites.

The three-dimensional platelets adhesion morphology of pure zinc and Zn-GNS composites are shown in Figure 2a. In contrast to the control group, most platelets adhered on pure zinc were round, without pseudopodia spreading and in an inactive state. It can be seen that pure zinc had no procoagulant effect after contacting blood. However, a small amount of pseudopodia protruding from the platelets on the surface of the Zn-GNS composites can be seen. It is worth noting that platelets adhere to all samples in a homogeneous way without aggregation. The results of the statistical analysis of the number of platelets adhered to the surface are shown in Figure 2b. The addition of GNS will not increase the number of platelets adhered to the surface. These results indicate that pure zinc and its composites have good antithrombotic and antiadhesion platelets properties.

The hemolysis rate of biomaterials in direct contact with blood must be less than 5% [6]. According to the hemolysis rate, materials can be divided into three different categories: materials with a hemolysis rate of more than 5% are hemolytic materials, between 2% and 5% are lightly hemolytic materials, and less than 2% are non-hemolytic materials. The percentage of hemolysis rate of pure zinc and Zn-GNS composites is showed in Figure 2c. The results showed that pure Zn and Zn-GNS composites were nonhemolytic with the hemolysis rate of less than 3%, suggesting a low risk of hemolysis. Therefore, pure zinc and Zn-GNS composites show good blood compatibility and can meet the requirements of biomaterials for hemolysis rate.

#### 3.2.2. Coagulation Time

**Figure 3:**
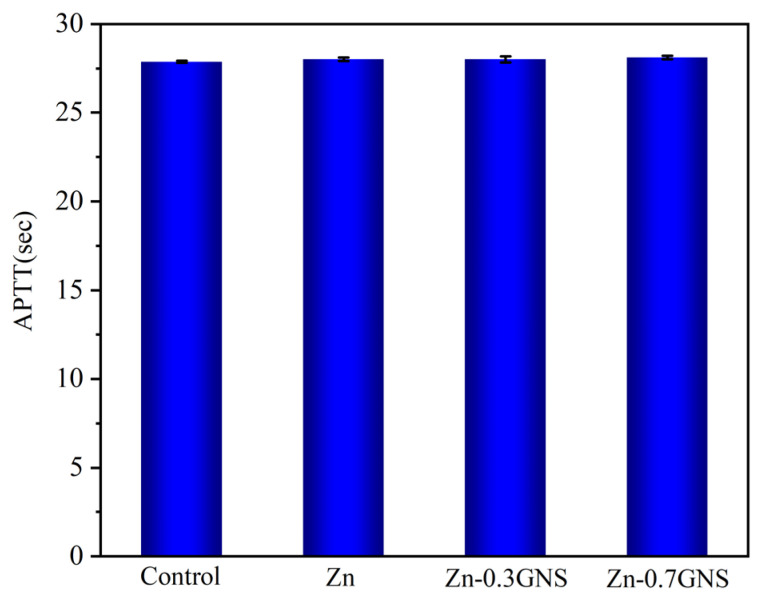
Coagulation time of pure Zn and Zn-GNS composites.

Activated partial thromboplastin time (APTT) is an important parameter for clinical blood tests and safety evaluation of biomaterials [36,37]. The effect of pure zinc and Zn-GNS composites on APTT are shown in Figure 3. Compared with the control group, the APTT of pure zinc and Zn-GNS composites has no significant change, indicating that all samples have better blood compatibility, which is consistent with the hemolysis test results. It has been reported that zinc alloy can prolong the activated partial thromboplastin time in vitro [38]. A similar phenomenon was also reported by Yang HT [10]. They studied the blood compatibility of Zn-xHA (0, 1, 5, 10 wt%) composites, and the results in terms of coagulation time showed that the APTT of Zn-10HA composites was prolonged from 37.37 s for the control to 58.15 s due to the higher zinc ion concentration. However, whether the APTT is dependent on zinc ion concentration needs to be further investigated.

### 3.3. Contact Angle

**Figure 4:**
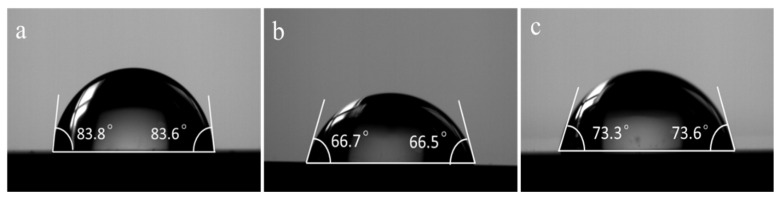
Contact angle images of (**a**) pure Zn, (**b**) Zn-0.3GNS, and (**c**) Zn-0.7GNS composites.

The wettability test results of pure Zn and Zn-GNS composites are shown in Figure 4. The contact angle of pure zinc is 82.8 ± 2.4° (Figure 4a), which is consistent with the contact angle (87°) of pure zinc prepared by microwave sintering process by Pathak et al. [39]. The contact angles of Zn-0.3GNS and Zn-0.7GNS are 66.4 ± 1.2° and 73 ± 2.9°, respectively. The results show that the addition of GNS will decrease the contact angle of the composites. It has been reported that the presence of oxygen-containing functional groups can improve the hydrophilicity of the composites [40]. GNS have been reported to have a large number of oxygen-containing functional groups in our previous study [27]. Therefore, the addition of GNS improves the hydrophilicity of Zn-GNS composites. However, when the GNS content was 0.7 wt%, the contact angle of the Zn-GNS composites instead increased, probably because of the strong van der Waals (vdW) interactions within the GNS layers promoting the agglomeration or stacking of the GNS layers, and higher concentrations of GNS are not uniformly dispersed in the metal matrix, which increased the contact angle. Solar et al. [41] reported that hydrophilic surfaces are more conducive to cell adhesion, while Li et al. [42] reported that cells tend to adhere to surfaces with a contact angle of about 70°. Therefore, the Zn-GNS composite exhibits better cell adhesion than pure Zn. In addition, the contact angles and hemolysis rates of the Zn-GNS composites fabricated in this study are listed in Table 1 and compared with the Zn-based materials reported in previous studies for biodegradable implant applications. The results showed that the Zn-GNS composites prepared in this study shows better blood compatibility and cell adhesion as compared to other zinc matrix materials.

## 4. Conclusions

This study analyzed the cell compatibility and blood compatibility of pure zinc and Zn-xGNS (0.3, 0.7 wt%) composites. Cytotoxicity tests showed that the Zn-GNS composites had acceptable toxicity to MG-63 cells. The results of hemocompatibility experiments show that the hemolysis rate of Zn-GNS composites is less than 3%, the platelets adhesion rate was low, and it has good antithrombotic properties. In addition, compared with pure zinc, the Zn-GNS composites have stronger hydrophilicity. Therefore, these research results showed that Zn-GNS composites have good biocompatibility. Zn-GNS composites show great application prospects as a new generation of biodegradable implants, providing a new research direction for the field of biodegradable metals.

## Figures and Tables

**Table 1:** Hemolysis rate and contact angle of various zinc matrix materials.

Materials	Contact Angle (°)	Hemolysis Rate (%)	Applications	Ref.
Zn (SPS + HR)	82.8 ± 2.4	1.527 ± 1.130	Orthopedic implants	this study
Zn-0.3GNS(SPS + HR)	66.4 ± 1.2	1.493 ± 0.089		
Zn-0.7GNS(SPS + HR)	73 ± 2.9	1.994 ± 0.691		
Zn (Cast)	-	2.083 ± 0.090	Orthopedic implants	[38]
Zn-1Fe (Cast)	-	3.750 ± 0.158		
Zn-2Fe (Cast)	-	4.583 ± 0.170		
Zn-5Fe (Cast)	-	17.50 ± 0.613		
Zn-10Fe (Cast)	-	20.83 ± 0.833		
Zn-1Mg (HR)	-	4.01 ± 0.03	Orthopedic implants	[30]
Zn-1Mg-0.1Er (HR)	-	4.49 ± 0.09		
Zn-1Mg-0.1Dy (HR)	-	4.21 ± 0.02		
Zn-1Mg-0.1Ho (HR)	-	3.28 ± 0.09		
Zn (MSP)	87.3	-	Biodegradable implants	[39]
Zn-3HA(MSP)	65.5	-		
Zn-3HA-2Fe (MSP)	77.3	-		
Zn-5HA-2Fe (MSP)	72.7	-		
Zn (AC)	62.4 ± 0.9	2.44 ± 0.08	Orthopedic implants	[2]
Zn-1Cu-0.1Ti (AC)	63.7 ± 2.6	3.62 ± 0.14		
Zn(Cast)	-	0.88 ± 0.07	Biodegradable implants	[43]
Zn-5Ge(Cast)	-	0.24 ± 0.1		
Zn(SPS)	-	1.07 ± 0.46	Orthopedic implants	[10]
Zn-1HA(SPS)	-	1.24 ± 0.78		
Zn-5HA(SPS)	-	0.63 ± 0.55		
Zn-10HA(SPS)	-	0.69 ± 0.44		
Zn(HE)	-	1.16 ± 0.39	Cardiovascular stents	[44]
Zn-3Cu(HE)	-	0.96 ± 0.48		
Zn-3Cu-0.2Fe(HE)	-	1.10 ± 0.25		
Zn-3Cu-0.5Fe (HE)	-	1.21 ± 0.27		

SPS: spark plasma sintering; HR: hot rolling; AC: air cooling; MSP: microwave sintering process; HE: hot extrusion.

## Data Availability

Not applicable.

## References

[1] Levy G.K., Goldman J., Aghion E. (2017). The prospects of zinc as a structural material for biodegradable implants—A review paper. Metals.

[2] Lin J., Tong X., Shi Z., Zhang D., Zhang L., Wang K., Wei A., Jin L., Lin J., Li Y. (2020). A biodegradable Zn-1Cu-0.1Ti alloy with antibacterial properties for orthopedic applications. Acta Biomater..

[3] Bommala V.K., Krishna M.G., Rao C.T. (2019). Magnesium matrix composites for biomedical applications: A review. J. Magnes. Alloy..

[4] Pierson D., Edick J., Tauscher A., Pokorney E., Bowen P., Gelbaugh J., Stinson J., Getty H., Lee C.H., Drelich J. (2012). A simplified in vivo approach for evaluating the bioabsorbable behavior of candidate stent materials. J. Biomed. Mater. Res. B..

[5] Yang H., Wang C., Liu C., Chen H., Wu Y., Han J., Jia Z., Lin W., Zhang D., Li W. (2017). Evolution of the degradation mechanism of pure zinc stent in the one-year study of rabbit abdominal aorta model. Biomaterials.

[6] Bowen P.K., Drelich J., Goldman J. (2013). Zinc Exhibits Ideal Physiological Corrosion Behavior for Bioabsorbable Stents. Adv Mater..

[7] Bowen P.K., Guillory R.J., Shearier E.R., Seitz J.M., Drelich J., Bocks M., Zhao F., Goldman J. (2015). Metallic zinc exhibits optimal biocompatibility for bioabsorbable endovascular stents. Mat. Sci. Eng. C-Mater..

[8] Meng Y., Liu L., Zhang D., Dong C., Yan Y., Volinsky A.A., Wang L.-N. (2019). Initial formation of corrosion products on pure zinc in saline solution. Bioact. Mater..

[9] Weiss J.H., Sensi S.L., Koh J.Y. (2000). Zn^2+^: A novel ionic mediator of neural injury in brain disease. Trends Pharmacol Sci..

[10] Yang H., Qu X., Lin W., Wang C., Zhu D., Dai K., Zheng Y. (2018). In vitro and in vivo studies on zinc-hydroxyapatite composites as novel biodegradable metal matrix composite for orthopedic applications. Acta Biomater..

[11] Suzuki T., Kajita Y., Katsumata S.-I., Matsuzaki H., Suzuki K. (2015). Zinc Deficiency Increases Serum Concentrations of Parathyroid Hormone through a Decrease in Serum Calcium and Induces Bone Fragility in Rats. J. Nutr. Sci. Vitaminol..

[12] Liu H., Li W., Jia S., Li B. (2017). Puerarin and zinc additively prevent mandibular bone loss through inhibiting osteoclastogenesis in ovariectomized rats. Histol. Histopathol..

[13] Kubásek J., Vojtěch D., Pospíšilová I., Michalcová A., Maixner J. (2016). Microstructure and mechanical properties of the micrograined hypoeutectic Zn-Mg alloy. Int. J. Min. Met. Mater..

[14] Erinc M., Sillekens W.H., Mannens R.G.T.M., Werkhoven R.J. (2009). Applicability of existing magnesium alloys as biomedical implant materials. Magnes. Technol..

[15] Tang Z., Huang H., Niu J., Zhang L., Zhang H., Pei J., Tan J., Yuan G. (2016). Design and characterizations of novel biodegradable Zn-Cu-Mg alloys for potential biodegradable implants. Mater. Design..

[16] Li H., Yang H., Zheng Y., Zhou F., Qiu K., Wang X. (2015). Design and characterizations of novel biodegradable ternary Zn-based alloys with IIA nutrient alloying elements Mg, Ca and Sr. Mater. Design..

[17] Paul W., Sharma C. (2011). Blood compatibility and biomedical applications of grapheme. Trends Biomater. Artif. Organs..

[18] Du X., Du W., Wang Z., Liu K., Li S. (2019). Defects in graphene nanoplatelets and their interface behavior to reinforce magnesium alloys. Appl. Surf. Sci..

[19] Zhou K., Yu P., Shi X., Ling T., Zeng W., Chen A., Yang W., Zhou Z. (2019). Hierarchically Porous Hydroxyapatite Hybrid Scaffold Incorporated with Reduced Graphene Oxide for Rapid Bone Ingrowth and Repair. ACS Nano.

[20] Yan J., Chen L., Huang C.-C., Lung S.-C.C., Yang L., Wang W.-C., Lin P.-H., Suo G., Lin C.-H. (2017). Consecutive evaluation of graphene oxide and reduced graphene oxide nanoplatelets immunotoxicity on monocytes. Colloid Surface B.

[21] Kumar S., Chatterjee K. (2015). Strontium eluting graphene hybrid nanoparticles augment osteogenesis in a 3D tissue scaffold. Nanoscale.

[22] Shahin M., Munir K., Wen C., Li Y. (2020). Magnesium-based composites reinforced with grapheme nanoplatelets as biodegradable implant materials. J. Alloys Compd..

[23] Shahin M., Munir K., Wen C., Li Y. (2019). Magnesium matrix nanocomposites for orthopedic applications: A review from mechanical, corrosion, and biological perspectives. Acta Biomater..

[24] Newman L., Lozano N., Zhang M., Iijima S., Yudasaka M., Bussy C., Kostarelos K. (2017). Hypochlorite degrades 2D graphene oxide sheets faster than 1D oxidised carbon nanotubes and nanohorns. NPJ 2D Mater. Appl..

[25] Munir K., Wen C., Li Y. (2020). Graphene nanoplatelets-reinforced magnesium metal matrix nanocomposites with superior mechanical and corrosion performance for biomedical applications. J. Magnes. Alloy..

[26] Saberi A., Bakhsheshi-Rad H., Karamian E., Kasiri-Asgarani M., Ghomi H. (2022). Magnesium-graphene nano-platelet composites: Corrosion behavior, mechanical and biological properties. J. Alloys Compd..

[27] Dai Q., Peng S., Zhang Z., Liu Y., Fan M., Zhao F. (2021). Microstructure and Mechanical Properties of Zinc Matrix Biodegradable Composites Reinforced by Graphene. Front. Bioeng. Biotech..

[28] (2009). Biological Evaluation of Medical Devices–Part 5: Tests for In Vitro Cytotoxicity.

[29] Tong X., Zhu L., Wang K., Shi Z., Huang S., Li Y., Ma J., Wen C., Lin J. (2022). Impact of gadolinium on mechanical properties, corrosion resistance, and biocompatibility of Zn-1Mg-xGd alloys for biodegradable bone-implant applications. Acta Biomater..

[30] Tong X., Zhang D., Lin J., Dai Y., Luan Y., Sun Q., Shi Z., Wang K., Gao Y., Lin J. (2020). Development of biodegradable Zn–1Mg–0.1RE (RE=Er, Dy, and Ho) alloys for biomedical applications. Acta Biomater..

[31] Lin J., Tong X., Sun Q., Luan Y., Zhang D., Shi Z., Wang K., Lin J., Li Y., Dargusch M. (2020). Biodegradable ternary Zn–3Ge–0.5X (X=Cu, Mg, and Fe) alloys for orthopedic applications. Acta Biomater..

[32] Murni N.S., Dambatta M.S., Yeap S.K., Froemming G.R.A., Hermawan H. (2015). Cytotoxicity evaluation of biodegradable Zn–3Mg alloy toward normal human osteoblast cells. Mat. Sci. Eng. C-Mater..

[33] Wang J., Witte F., Xi T., Zheng Y., Yang K., Yang Y., Zhao D., Meng J., Li Y., Li W. (2015). Recommendation for modifying current cytotoxicity testing standards for biodegradable magnesium-based materials. Acta Biomater..

[34] Oba Y., Yasue A., Kaneko K., Uchida R., Shioyasono A., Moriyama K. (2008). Comparison of stability of mandibular segments following the sagittal split ramus osteotomy with poly-l-lactic acid (PLLA) screws and titanium screws fixation. Orthod. Waves.

[35] Lee S.R. (2018). Critical role of zinc as either an antioxidant or a prooxidant in cellular systems. Oxid. Med. Cell Longevity..

[36] Wang J., He Y., Maitz M.F., Collins B., Xiong K., Guo L., Yun Y., Wan G., Huang N. (2013). A surface-eroding poly (1,3-trimethylene carbonate) coating for fully biodegradable magnesium-based stent applications: Toward better biofunction, biodegradation and biocompatibility. Acta Biomater..

[37] Mochizuki A., Kaneda H. (2015). Study on the blood compatibility and biodegradation properties of magnesium alloys. Mat. Sci. Eng. C-Mater..

[38] Králová Z.O., Gorejová R., Oriňaková R., Petráková M., Oriňak A., Kupková M., Hrubovčáková M., Sopčák T., Baláž M., Maskaľová I. (2021). Biodegradable zinc-iron alloys: Complex study of corrosion behavior mechanical properties and hemocompatibility. Prog. Nat. Sci.-Mater..

[39] Pathak D.K., Pandey P.M. (2021). Evaluation of in vitro corrosion behavior of zinc–hydroxyapatite and zinc–hydroxyapatite–iron as biodegradable composites. J. Biomed. Mater. Res. B Appl. Biomater..

[40] Abazari S., Shamsipur A., Bakhsheshi-Rad H.R., Berto F. (2022). Functionalized carbon nanotube-encapsulated magnesium-based nanocomposites with outstanding mechanical and biological properties as load-bearing bone implants. Mater. Design..

[41] Solař P., Kylián O., Marek A., Vandrovcová M., Bačáková L., Hanuš J., Vyskočil J., Slavínská D., Biederman H. (2015). Particles induced surface nanoroughness of titanium surface and its influence on adhesion of osteoblast-like MG-63 cells. Appl. Surf. Sci..

[42] Li Q., Wang Z., Zhang S., Zheng W., Zhao Q., Zhang J., Wang L., Wang S., Kong D. (2013). Functionalization of the surface of electrospun poly(epsilon-caprolactone) mats using zwitterionic poly (carboxybetaine methacrylate) and cell-specific peptide for endothelial progenitor cells capture. Mat. Sci. Eng. C-Mater..

[43] Tong X., Zhang D., Zhang X., Su Y., Shi Z., Wang K., Lin J., Li Y., Lin J., Wen C. (2018). Microstructure, mechanical properties, biocompatibility, and in vitro corrosion and degradation behavior of a new Zn–5Ge alloy for biodegradable implant materials. Acta Biomater..

[44] Yue R., Niu J., Li Y., Ke G., Huang H., Pei J., Ding W., Yuan G. (2020). In vitro cytocompatibility, hemocompatibility and antibacterial properties of biodegradable Zn-Cu-Fe alloys for cardiovascular stents applications. Mat. Sci. Eng. C-Mater..

